# Association between perinatal factors, genetic susceptibility to obesity and age at adiposity rebound in children of the EDEN mother–child cohort

**DOI:** 10.1038/s41366-021-00847-w

**Published:** 2021-05-13

**Authors:** Aminata Hallimat Cissé, Sandrine Lioret, Blandine de Lauzon-Guillain, Anne Forhan, Ken K. Ong, Marie Aline Charles, Barbara Heude

**Affiliations:** 1 Université de Paris, CRESS, INSERM, INRAE, F-75004, Paris, France; 2 MRC Epidemiology Unit and Department of Paediatrics, Institute of Metabolic Science, University of Cambridge, Cambridge, UK

**Keywords:** Public health, Epidemiology

## Abstract

**Background:**

Early adiposity rebound (AR) has been associated with increased risk of overweight or obesity in adulthood. However, little is known about early predictors of age at AR. We aimed to study the role of perinatal factors and genetic susceptibility to obesity in the kinetics of AR.

**Methods:**

Body mass index (BMI) curves were modelled by using mixed-effects cubic models, and age at AR was estimated for 1415 children of the EDEN mother–child cohort study. A combined obesity risk-allele score was calculated from genotypes for 27 variants identified by genome-wide association studies of adult BMI. Perinatal factors of interest were maternal age at delivery, parental education, parental BMI, gestational weight gain, maternal smoking during pregnancy, and newborn characteristics (sex, prematurity, and birth weight). We used a hierarchical level approach with multivariable linear regression model to investigate the association between these factors, obesity risk-allele score, and age at AR.

**Results:**

A higher genetic susceptibility to obesity score was associated with an earlier age at AR. At the most distal level of the hierarchical model, maternal and paternal educational levels were positively associated with age at AR. Children born to parents with higher BMI were more likely to exhibit earlier age at AR. In addition, higher gestational weight gain was related to earlier age at AR. For children born small for gestational age, the average age at AR was 88 [±39] days lower than for children born appropriate for gestational age and 91 [±56] days lower than for children born large for gestational age.

**Conclusion:**

The timing of AR seems to be an early childhood manifestation of the genetic susceptibility to adult obesity. We further identified low birth weight and gestational weight gain as novel predictors of early AR, highlighting the role of the intrauterine environment in the kinetics of adiposity.

## Introduction

After birth, body mass index (BMI) increases to a peak during the first year of life and declines between age 4 and 6 years, then increases again. This BMI increase following the minimum is called adiposity rebound (AR) [1–3]. This period has been characterized by cessation in the decline of fat mass index (defined as fat mass divided by height square) [4]. The timing of AR, independent of BMI itself, is considered a predictor of obesity in later childhood and adulthood [5–7]. More specifically, early AR is associated with increased risk of overweight or obesity and cardiometabolic diseases in adulthood [8–10].

To provide a track to prevent the development of obesity, one must follow children’s BMI growth curve and better understand the determinants of age at AR. Furthermore, the DoHAD theory [11] suggests that the intrauterine and early postnatal environment are crucial to the child’s and adult’s healthy development [12], along with the existence of a nutritional and/or metabolic programming of the development of obesity [13]. As an example, excessive and insufficient gestational weight gain was associated with increased risks of adverse childhood outcomes for the offspring such as low and high birth weight [14] and obesity in later life [15, 16]. Similarly, several studies have shown being small or large for gestational age (LGA) associated with increased risk of overweight and obesity [17, 18]. Although the link between intrauterine and early postnatal environment factors and age at AR has not been thoroughly investigated in the literature, a set of early prenatal and postnatal determinants of age at AR have been identified. A longitudinal study interested in pre-, perinatal, and parental determinants of the kinetics of the BMI curve among 1681 children showed that girls had earlier age at AR than boys [19]. Parental educational level, especially low maternal level of education (<7 years), is an additional important risk factor for early AR [2]. In their retrospective study, Péneau et al. examined the determinants of age at AR and reported that a larger maternal silhouette was associated with early age at AR in both boys and girls, while the paternal silhouette was related with early age at AR in girls only [20]. However, further evidence is needed to confirm these findings with measured parental weight data instead of reported silhouettes.

Genome-wide associations studies have highlighted single-nucleotide polymorphisms (SNPs) implicated in increased risk of obesity [21, 22] and the use of genetic susceptibility scores has been established. In a meta-analysis of four European cohorts [23], Elks et al. showed that association with this genetic susceptibility score emerges progressively associated in childhood with a child’s weight, height, and BMI. However, to our knowledge, only one study has investigated the role of this genetic susceptibility to obesity in the kinetics of BMI at the time of AR [24].

In this context, the purpose of this study was to investigate the role of parental education, perinatal factors, and genetic susceptibility to obesity in the kinetics of AR within the French EDEN mother–child cohort. We hypothesize that the association between age at AR and perinatal factors is independent of genetic susceptibility to obesity and that age at AR could be programmed by a set of factors involved as early as in pregnancy.

## Material and methods

### Study population

The EDEN mother–child study is a prospective French cohort that aims to assess prenatal and postnatal determinants of child growth, health, and development. This cohort included 2002 pregnant women (before 24 weeks of amenorrhea) recruited from 2003 to 2006 in two university hospitals in Nancy and Poitiers. Exclusion criteria were <18 years of age, multiple pregnancies, known diabetes before pregnancy, illiteracy, and intention to give birth elsewhere than these two French hospitals or to move outside the region within the next 3 years. Details of the study protocol have been published elsewhere [25].

The study was approved by the relevant ethical research Committee of Kremlin‐Bicêtre Hospital and by the Data Protection Authority. Both parents gave their written consent.

### Data collection and variables generation

#### Data collection

Data were collected from medical and obstetrical records or by interviews, face-to-face or self-administered questionnaires and clinical examinations. Biological samples, anthropometric, and clinical parameters measurements were collected during clinical examinations at birth, 1, 3, and 5 years. In all children, weight and height from birth to early adolescence were obtained during these visits or from health records. Children had a mean of 10 weight and height measurements (interquartile range 6–14 and 5–13, respectively) from birth to 13 years. Maternal smoking status during pregnancy (yes/no); parental characteristics such as maternal age (years), maternal and paternal educational level and parental anthropometric measurements were collected during pregnancy. Newborn characteristics as well as sex, gestational age, and birth weight were taken at birth [26]. Preterm birth (yes/no) was defined as babies born before 37 weeks of gestation. Birth weight customized *z*-scores were calculated according to Gardosi references taking into account physiological fetal (sex and gestational age) and maternal factors (weight, height, parity, origin) [27]. Newborns were classified, according to this customized approach, into three classes: small for gestational age (SGA, ≤10th percentile), appropriate for gestational age (AGA, >10th percentile to ≤90^th^ percentile), and LGA (>90^th^ percentile).

Maternal and paternal education level was considered as the number of years of study. Parental weight and height were collected by interview at inclusion. Parental anthropometric measurements were collected at inclusion were used to calculate parental BMI by dividing the weight (kilograms) by the square of height (meters). Gestational weight gain (kilograms) was collected from obstetric records.

#### Genotyping and BMI obesity risk-allele score

DNA samples were extracted from cord-blood at birth. Genotyping for 27 single-nucleotide polymorphisms (SNPs) was performed by the Epidemiology Unit of the Medical Research Council of Cambridge (iPLEX platform; Sequenom) [28]. Genotyping quality-control criteria (call rate, >95%; Hardy Weinberg balance, *p* > 0.01) were satisfactory for all variants. In the present study, we considered 27 of 32 SNPs associated with BMI in adults [21], of which also the 16 associated with childhood BMI in a meta-analysis of Elks et al. [23].

For each child, combined obesity risk‐allele scores across the 27 SNP loci were calculated as the sum of risk alleles (0, 1, or 2 at each locus) associated with increased BMI. The score indicating genetic predisposition to obesity ranged from 11 to 32. Combined obesity risk‐allele scores were available for 1322 children of the EDEN cohort and their mothers (*N* = 1678) and fathers (*N* = 1241) [29].

A weighted obesity risk-allele score was also computed by multiplying each risk-allele by the effect estimate on adult BMI as assessed in the genome-wide association study [21], for sensitivity analyses.

#### Estimation of AR

Age at AR is defined as the last minimum (nadir) BMI before the continuous increase in BMI over time [1].

We calculated BMI from weight and height measurements. We excluded children with fewer than three BMI measurements from age 18 months to 13 years. Included children had 10 BMI values on average (interquartile range 6–14) from age 18 months to 13 years. Individual growth curves were obtained by using mixed-effects cubic models separately for girls and boys. The methods for growth modeling of age at AR were inspired by the model of Sovio et al. [30]. To this previously described model, we added random effects for the quadratic and cubic terms for age with unstructured covariance matrix, which provided the best fit of individual predicted curves. The model was then fitted for log-transformed BMI, separately for boys and girls, as follows:

$$\log \left( {{\mathrm{BMI}}} \right) = \beta _0 + \mu _0 + (\beta _1 + \mu _1){\mathrm{Age}} + (\beta _2 + \mu _2){\mathrm{Age}}^2 + (\beta _3 + \mu _3){\mathrm{Age}}^3 + \varepsilon$$

where BMI is expressed in kilograms per square meter and age in days; *β*_0_, *β*_1,_ *β*_2_, *β*_3_, and *β*_4_ are the fixed-effects terms; *μ*_0,_ *μ*_1,_ *μ*_2_, and *μ*_3_ are the individual-level random effects; and *ɛ* is the residual.

For each participant, we estimated age at AR by the first and second derivatives of individual BMI functions. Results of this modeling process are illustrated for a random sample of ten participants in Supplementary Fig. S1.

### Sample selection

**Fig. 1:**
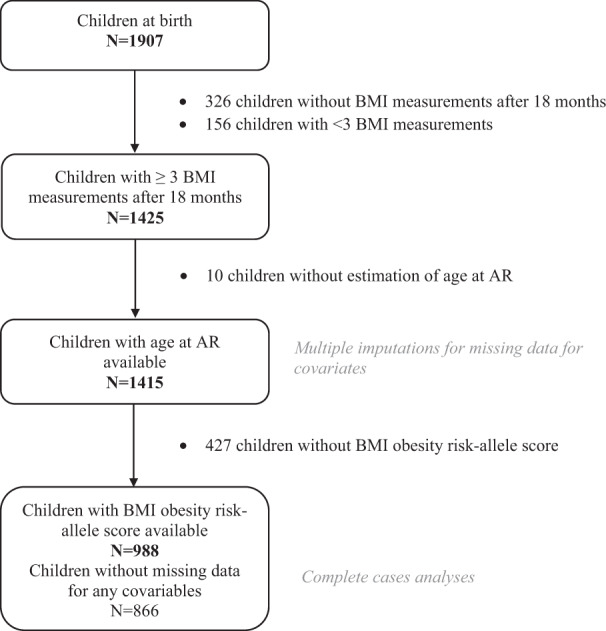
Flow chart of the population included in the study.

Among the 1907 newborns included in the EDEN cohort, we excluded 326 children without BMI measurements after 18 months and a further 126 who had fewer than three BMI measurements between age 18 months and 13 years. We also excluded ten children for whom AR could not be assessed because their BMI curve did not display any AR (see Supplementary Fig. S2). Therefore, the final sample included 1415 children; 69.8% (988) had available obesity risk-allele scores (Fig. 1).

### Statistical analysis

Main characteristics of study population were described with mean ± SD or percentage (*N*) before any imputation. We tested differences between included and excluded populations with chi-square tests for categorical variables and Student *t* for continuous variables. We also described and compared covariates according to each quintile of age at AR by chi-square tests and ANOVA. The associations between perinatal factors, obesity risk-allele score, and age at AR were investigated by linear multiple regression models.

By using the predictors identified in the literature, we developed a hierarchical level approach to answer our research question (Supplementary Fig. S3), which assumes that proximal variables have more direct effect than distal variables: the relevance of a factor is determined in the model in which the variable was first entered, without considering its performance in other models. This approach allows us to prevent that intermediate variables affect the association between distal variables and the dependent variable.

Therefore, we tested four successive nested models. Model A included the most distal-level variables corresponding to demographic and socioeconomic factors (maternal age at delivery, maternal and paternal educational level), along with recruitment center; model B further included factors related to obesity risk such as child’s obesity risk-allele score and maternal and paternal BMI; model C included also variables corresponding to the intrauterine and prenatal environment (gestational weight gain and maternal smoking during pregnancy); model D corresponded to the most proximal level, with the inclusion of newborn characteristics (sex, prematurity, and birth weight *z*-score). Birth weight *z*-score was introduced with a squared term to test for a potential quadratic shape of the association.

To assign a value to missing data for covariates (Supplementary Table S1), we first performed multiple imputation analyses, assuming that data were missing at random. Using the fully conditional specification method, we generated 40 independent datasets. Then, we computed pooled effect estimates. Parental obesity risk-allele scores were used to impute children’s scores when these were missing. With multiple imputation, the main analysis involved a sample of 1415 children.

We conducted a first sensitivity analysis running our multivariable models with data for the 866 children with complete cases. Most of the decline in sample size was due to the lack of obesity risk-allele score for many children. As a second sensitivity analysis, we tested the associations between weighted obesity risk-allele score, perinatal determinants, and age at AR in the fully adjusted model with multiple imputation. Finally, we conducted a sensitivity analysis excluding children born prematurely.

As complementary analyses, we first performed association analyses between perinatal factors, obesity risk-allele score, and risk of early AR, using logistic multiple regression models, estimating odds ratios and 95% confidence intervals (OR [95% CI]). We defined early AR as a rebound occurring before the first quintile of the distribution of age at AR. Then, we investigated each polymorphism used to establish the obesity risk-allele score and tested its individual association with age at AR. For this analysis, we assumed that the alleles of a given SNP have co-dominant effects, as is the case for the score computation. Therefore, we considered the SNPs (categorized as 0, 1, and 2) as continuous variables. The analysis of each SNP involved linear regression analysis of complete cases with *β* corresponding to the change in days for one additional mutated allele carried.

We considered nominal *p* < 0.05 as statistically significant. We used R v3.5.1 for modeling BMI trajectories and estimating age at AR and SAS v9.4 (SAS, Cary, NC, on an AIX 7.1 platform) for all other analyses.

## Results

### Population characteristics

Compared to mothers of excluded children, those of included children (*n* = 1415) had on average a higher number of years of study and were older at delivery (*p* = 3.0 × 10^−10^) (Table 1). Included infants were born at higher gestational age (*p* = 0.03) and had higher birth weight (*p* = 0.03) than excluded children but were similar in sex (*p* = 0.79) and maternal pregestational BMI (*p* = 0.08).

**Table 1:** Characteristics of the included and excluded EDEN cohort in the study of adiposity rebound.

	Mean (SD) or % (*N*)		*p* > |*t*| or *χ*^2^
	Included	Excluded	
	*N* = 1415	*N* = 492	
Sex (boys)	52.7 (746)	52.1 (254)	0.79
Preterm birth	5.5 (78)	6.5 (32)	0.41
Birth weight (kg)	3.29 (0.5)	3.23 (0.5)	0.03
Birth weight *z*-score			0.04
SGA	13.3 (184)	13.2 (62)	
AGA	77.4 (1070)	81.3 (383)	
LGA	9.3 (128)	5.5 (26)	
Gestational weight gain (kg)	13.3 (4.7)	13.8 (5.2)	0.03
Maternal BMI (kg/m^2^)	23.1 (4.4)	23.6 (5.1)	0.08
Paternal BMI (kg/m^2^)	25.1 (3.6)	24.7 (3.7)	0.03
Never smoking during pregnancy	78.6 (1088)	59.4 (275)	4.0 × 10^−16^
Maternal age at delivery (years)	29.9 (4.7)	28.2 (5.2)	3.0 × 10^−10^
Maternal educational level (years)	14.0 (2.6)	12.5 (2.5)	4.5 × 10^−30^
Paternal educational level (years)	13.3 (2.6)	12.2 (2.4)	3.7 × 10^−18^
Obesity risk‐allele score	21.6 (3.2)	21.3 (3.4)	0.15
Age at adiposity rebound (years)			0.08^a^
Boys	5.5 (1.3)	–	–
Girls	5.4 (1.4)	–	–

*BMI* body mass index.

^a^*p* value of mean age at adiposity rebound comparing boys and girls.

Among the 1415 children included, the mean (±SD) age of AR was estimated at 5.5 (±1.3) and 5.4 (±1.4) years for boys and girls, respectively (*p* = 0.08). The first quintile of the distribution of age at AR was estimated at 4.4 years.

The description of predictors according to the quintiles of age at AR is provided in Supplementary Table S2. Except for prematurity, gestational weight gain, maternal age at delivery, and obesity risk-allele score, all considered factors were significantly differed by quintile of age at AR.

### Determinants associated with the timing of AR

Multivariable associations with age at AR are displayed in Table 2. Model A showed that high maternal and paternal educational level were associated with later AR (*β* [±SE] = 15.7 [±6.2] and 14.4 [±6.0] days per year of study, *p* = 0.01 and 0.02, respectively), with no association with the other socio-demographic factors considered in this model. Furthermore, all variables related to parental obesity (Model B) and prenatal and intrauterine factors (Model C) were significantly associated with age at AR: age at AR decreased with mean obesity risk-allele score (−9.2 [±4.6] days per allele, *p* = 0.05), maternal and paternal BMI (−15.8 [±3.1] days per kg/m^2^, *p* = 1.9 × 10^−7^, and −15.6 [±3.9] days per kg/m^2^, *p* = 5.6 × 10^−5^), gestational weight gain (−7.5 [±2.9] days per kg, *p* = 0.001) and smoking during pregnancy (−72.1 [±32.6] days, *p* = 0.03). Finally, birth weight *z*-score was the only child characteristic at birth associated with age at AR with a significant quadratic effect (*p* = 0.05).

**Table 2:** Multivariable linear regression of factors associated with age at adiposity rebound (*N* = 1415^a^).

Characteristics	Model A		Model B		Model C		Model D	
	*β* (SE)	*p* value	*β* (SE)	*p* value	*β* (SE)	*p* value	*β* (SE)	*p* value
Level 1^b^								
Center (ref = Poitiers)	−0.8 (26.6)	0.98	−14.1 (26.2)	0.59	−22.3 (26.2)	0.40	−14.9 (26.3)	0.57
Maternal age at delivery (years)	2.8 (2.9)	0.33	5.1 (2.8)	0.070	3.7 (2.9)	0.19	3.9 (2.8)	0.17
Maternal educational level (years)	15.7 (6.2)	0.011	13.4 (6.1)	0.027	11.3 (6.1)	0.064	10.0 (6.1)	0.10
Paternal educational level (years)	14.4 (6.0)	0.017	8.5 (6.0)	0.16	7.4 (6.0)	0.22	7.0 (6.0)	0.24
Level 2								
Obesity risk-allele score			−9.2 (4.6)	0.045	−9.1 (4.6)	0.045	−9.0 (4.5)	0.047
Maternal BMI (kg/m^2^)			−15.9 (3.1)	1.9 × 10^−7^	−18.2 (3.1)	6.3 × 10^−9^	−18.0 (3.1)	8.9 × 10^−9^
Paternal BMI (kg/m^2^)			−15.6 (3.9)	5.6 × 10^−5^	−15.3 (3.9)	6.9 × 10^−5^	−15.3 (3.8)	6.7 × 10^−5^
Level 3								
Gestational weight gain (kg)					−7.5 (2.9)	0.009	−8.6 (3.0)	0.004
Smoking during pregnancy (ref = no)					−72.1 (32.6)	0.027	−62.3 (32.7)	0.057
Level 4								
Preterm birth (yes vs no)							39.4 (57.7)	0.50
Birth weight *z*-score							24.1 (12.4)	0.053
Birth weight *z*-score [2]							−14.4 (7.3)	0.047
Sex (girls vs boys)							−47.2 (25.8)	0.067

^a^Missing data for covariates were imputed with the fully conditional specification method.

^b^Relevance of a variable during the hierarchical regression analyses was determined with the corresponding model in which the variable of interest is first entered, regardless of its performance in the subsequent model(s).

**Fig. 2:**
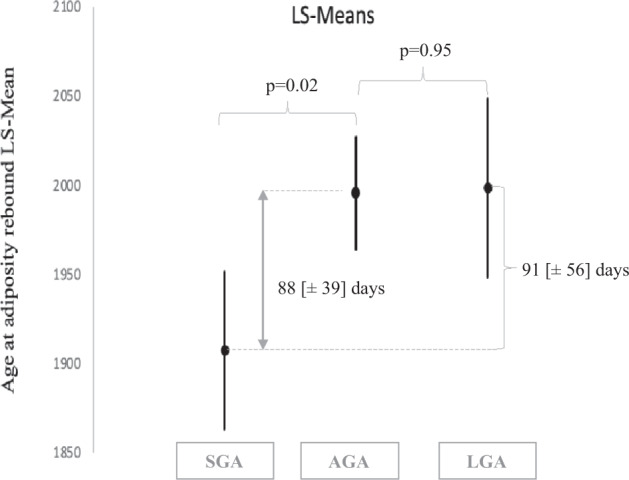
Adjusted means [SE] of age at adiposity rebound according to birth weight *z*-score categories (*N* = 1415^a^, model D^b^). ^a^Missing data for covariates were imputed with the fully conditional specification method. ^b^Adjusted for center, maternal age at delivery, maternal and paternal educational level, maternal and paternal BMI, gestational weight gain, smoking duringpregnancy, prematurity, birth weight *z*-score and sex.

To illustrate this last result, the mean age of AR and their confidence interval by category of birth weight *z*-score are presented in Fig. 2. On average, children born SGA had an age at AR 88 [±39] days lower than children born AGA, and 91 [±56] days earlier than children born LGA.

### Sensitivity and complementary analyses

In the sensitivity analysis, results were on the whole consistent with analyses limited to children with no missing data (complete-case analysis) (Supplementary Table S3). However, the associations observed between maternal educational level (Model A), smoking during pregnancy (Model C) and age at AR were no longer significant. We also observed that the strength of association was higher with obesity risk-allele score and birth weight *z*-score, but lower with parental BMI. Results with the weighted obesity risk-allele score were consistent with, albeit slightly weaker, than those with the unweighted obesity risk-allele score (Supplementary Table S4). When preterm children were removed from the analyses, the association between birth weight *z*-score and age at AR became stronger and the risk of early AR for SGA was greater (2.23 [1.52–3.28]) (Supplementary Table S5).

The results of logistic regression analyses testing the association between obesity risk-allele score, perinatal factors, and odds of early AR, are summarized in Supplementary Fig. 4. Higher maternal educational level was associated with reduced odds of early AR. Odds of early AR were increased with each additional obesity risk-allele, higher maternal and paternal BMI, and higher gestational weight gain. Risk of early AR was higher for children born SGA than AGA children. We found no association between LGA children and risk of early AR. Risk of early AR was higher for girls than boys.

In analyses considering each polymorphism separately (Supplementary Fig. S5), we found no significant association. The associations between rs10913469 at the locus harboring SEC16B, rs713586 at the locus harboring RBJ/POMC and age at AR were at the limit of significance.

## Discussion

We found that genetic susceptibility to obesity was negatively associated with age at AR in the EDEN mother–child study. Additionally, we confirmed that some previously described perinatal factors, such as parental BMI, were associated with age at AR [2, 20] but also further identified novel predictors such as birth weight and mother’s gestational weight gain. Probability of early AR was increased with high gestational weight gain and low birth weight.

This is the first study to simultaneously analyze the association between perinatal factors, genetic predisposition to obesity and age at AR, taking the assumed hierarchical structure between the potential predictors into account. At the most distal level, maternal and paternal education were negatively associated with risk of early AR, in accordance with previous studies [2]. This association was attenuated with the inclusion of the more proximal groups of variables. A longitudinal study of 889 representative children in the United Kingdom showed no association between parental educational level or socioeconomic factors and time of AR [31]. This result was obtained after adjustment for parental BMI, which make our two studies concordant. Future studies could use proper mediation analyses to quantify the mediating contribution of each of these factors and help prioritize effective interventions to reduce social inequalities in obesity.

The genetic susceptibility to obesity was associated with an increased probability of early AR. The role of genetic factors in AR timing was investigated recently in a genome-wide association study including up to 7215 children in five European cohorts. In this study, Couto Alves et al. identified an association between two SNPs and age at AR. Although the rs1421085 (at the locus harboring *FTO*) was not associated with age at AR, we noted a statistically significant association for rs10913469 (at the locus harboring SEC16B) and an association at the limit of significance for rs713586 (at the locus harboring RBJ/POMC) but with reduced statistical power. In accordance with our results, the authors reported a negative association between an obesity risk-allele score, based on 97 SNPs, and child age at AR, but did not model the effects of other life factors [24]. Altogether, these results illustrate that the observed association between a score of genetic susceptibility to obesity and timing of AR is the result of a cumulative effect of individual SNPs. The authors also highlighted the relevance of using cumulative scores to address the genetic predisposition to early AR.

Consistent with previous research, we found that high parental BMI predicted early AR in children. A longitudinal study of 248 families enrolled in North Carolina concluded high maternal BMI as the robust predictor of early AR [2]. Another longitudinal study of a New Zealand cohort found that mothers and fathers of children with early BMI rebound had significantly heavier as compared with late rebounders [32]. Two other investigations also demonstrated that children with early AR had at least one parent with obesity [31, 33]. All these analyses recognized maternal BMI as a proxy variable reflecting the child’s susceptibility to obesity. However, we found the association between parental BMI and age at AR was independent of the obesity risk-allele score, which suggests that parental BMI might have an effect independent of genetic predisposition to obesity. Indeed, parental BMI also reflects an obesogenic environment (familial environment, food choices, eating patterns, and physical activity) that may affect the kinetics of adiposity during childhood [34, 35]. Moreover, we observed that the association of mother’s prepregnancy BMI seemed to be slightly higher, even if not significantly (results not shown), than that of paternal BMI, which also suggests a role for nutritional environment during prenatal life.

Our results regarding gestational weight gain support this latter hypothesis. To our knowledge, only one study has considered gestational weight gain as a potential predictor of age at AR, but no significant association was shown [36]. However, previous reports focused on the link between gestational weight gain and childhood overweight at various ages. In the current EDEN cohort, a positive association was found between gestational weight gain and child’s BMI at age 5–6 years [37]. Likewise, a meta-analysis of 37 cohort studies from Europe, North America, and Australia showed that high gestational weight gain was related with increased risk of overweight/obesity in childhood, with stronger associations effects observed at later ages [15]. Our results suggest that the age of AR could in part mediate the association between gestational weight gain and overweight/obesity in late childhood and adolescence described in this meta-analysis, but further research would be necessary to confirm this assumption.

We found a negative association between maternal smoking status during pregnancy and the timing of AR but not risk of early AR. A longitudinal analysis, based on mothers from the longitudinal Growth and Obesity Chilean Cohort Study, also showed no link between smoking status during pregnancy and risk of early AR [38]. In previous analyses of the EDEN cohort, Carles et al. applied a joint Bayesian model to explore the association between maternal smoking status during pregnancy and growth [39]: children from mothers who smoked throughout pregnancy were born smaller but had higher BMI in their first months of life until age 5 as compared with children born to non-smokers. In the Generation R study, maternal smoking during pregnancy was associated with increased BMI at age 4 years for children with normal birth weight and SGA at birth [40]. Conversely, in the ALSPAC cohort, smoking during pregnancy was not associated to child weight status at age 5 years [41]. Thus, the association between maternal smoking during pregnancy and rapid postnatal growth might be due to its impact on fetal development but also to confounding by socioeconomic factors. Further studies are needed to confirm or not any effect of smoking during pregnancy on age at AR.

For the first time, we identified birth weight for gestational age as a predictor of age at AR. Some previous studies did include birth weight in their multiple models but did not find any significant association with age at AR. For the “Children in Focus” group from the ALSPAC cohort, no difference in birth weight was observed between the groups of very early, early, and later AR [42]. In contrast, our results indicate that children born SGA were at increased risk of early AR, whereas children born LGA were not. A cross-sectional study in Lausanne showed an association between low birth weight and anthropometric obesity markers in adulthood [43]. Similarly, the review article of Hong and Chung highlighted the association between SGA children and greater adiposity and obesity in later life [17]. However, Kapral et al. found this association only in preterm children [18]. These findings highlight that SGA newborns tend to rapidly “catch up”, and that this “catch-up” is characterized by an increase in fat mass greater than lean mass [44]. Furthermore, several systematic reviews have shown a strong association between rapid infancy weight gain and greater risk of obesity, overweight or increased adiposity in later life [45, 46]. The catch-up phenomenon would be due to different mechanisms of adaptation of a newborn exposed to undernutrition in utero, which could have remote impact on age at AR [47]. The respective role of intrauterine growth restriction vs postnatal growth acceleration in this association still needs to be disentangled.

Even if some of our results need confirmation and replication, they highlight a number of prenatal factors, including smoking during pregnancy, maternal prepregnancy BMI, gestational weight gain and birth weight for gestational age, possibly involved in the timing of AR. These findings strongly suggest that some intrauterine programming may also be at work in the etiology of this specific adiposity phenotype. Among the possible mechanisms, altered fetal nutrition could lead to persistent changes in the structure and function of adipose tissue, regulation of appetite and energy metabolism, which could affect the kinetics of adiposity and its rebound [15]. Epigenetic mechanisms could also be involved, but further studies are needed to better understand these [48].

The main strength of this study is that anthropometric data were assessed repetitively and prospectively from birth to age 13, which allowed us to estimate age at AR for a large number of children. As is often the case in cohort studies, the presence of selection bias at inclusion and missing data of covariates but also attrition bias limits the generalization of the results. However, multiple imputations and growth modeling of individual growth curves helped reduce the risk of these biases. Another limitation is that we could not consider ethnicity in our analyses, because the information was not collected in the EDEN cohort. Nonetheless, for 85% of children in the EDEN cohort, both parents were born in France and came from French regions where most of the population has a Caucasian origin. Moreover, the construction of the genetic score relies on the assumption of a co-dominant effect of each allele of a given SNP, which is not always true and could induce measurement errors. Our results, relying on observational data, need to be replicated in other studies with larger samples and with distinct designs to provide additional arguments for a causal interpretation of the observed associations. Further studies are needed to examine postnatal factors involved in the kinetics of BMI growth curve, to better characterize children at risk of early rebound. Indeed, the detection of early age at AR might be a useful tool for tailored and personalized child’s growth monitoring aiming at preventing overweight/obesity and chronic disease in later life.

## Conclusion

We confirmed that genetic susceptibility to adult obesity plays a predictive role in the timing of age at AR. Timing of AR in early childhood may contribute to the path from genetic susceptibility to obesity onset. Independent of this genetic background, we identified several prenatal predictors, including two novel factors, gestational weight gain and birth weight. These results strongly support that intrauterine programming may also be at work in the etiology of this specific adiposity phenotype.

## Supplementary information

Table S1, Table S2, Table S3, Table S4, Table S5, Figure S1, Figure S2, Figure S3, Figure S4, Figure S5
