# Supplementary material for: Association between perinatal factors, genetic susceptibility to obesity and age at adiposity rebound in children of the EDEN mother–child cohort
Source: Int J Obes (Lond). 2021 May 13;45(8):1802–10. doi: 10.1038/s41366-021-00847-w (PMC8310796; doi:10.1038/s41366-021-00847-w)
Supplement: Supplementary file 1 — Table S1, Table S2, Table S3, Table S4, Table S5, Figure S1, Figure S2, Figure S3, Figure S4, Figure S5 [file 41366_2021_847_MOESM1_ESM.docx]

**Supplementary table S1: Details of the method used for multiple imputation**

| Variables | Type of variable | Model used to predict missing data* | Missing value (%) |
| --- | --- | --- | --- |
| Age at adiposity rebound | Continuous |  | 0 |
| Early adiposity rebound | Binary | Logistic regression |  |
| Center | Binary | Logistic regression | 0 |
| Sex | Binary | Logistic regression | 0 |
| Birth weight | Continuous | Linear regression | 0 |
| Personalized birth weight z-score | Continuous | Linear regression | 2.3 |
| Gestationnal age | Continuous | Linear regression | 0 |
| Preterm birth | Binary | Logistic regression | 0 |
| Weigh predicted at 5 years by Jenss-Bayley modelling** | Continuous | Linear regression | 0 |
| Height predicted at 5 years by Jenss-Bayley modelling** | Continuous | Linear regression | 0 |
| Child’s obesity risk-allele score | Continuous | Linear regression | 30.4 |
| Maternal age at delivery | Continuous | Linear regression | 0 |
| Gestational weight gain | Continuous | Linear regression | 1.9 |
| Smoking during pregnancy | Binary | Logistic regression | 2.2 |
| Maternal education | Continous | Linear regression | 0 |
| Maternal income | Ordinal (3 categories) | Multinomial regression | 0.6 |
| Maternal BMI | Continuous | Linear regression | 1.8 |
| Maternal obesity risk-allele score | Continuous | Linear regression | 11.7 |
| Paternal education | Continous | Linear regression | 0 |
| Paternal BMI | Continuous | Linear regression | 6.7 |
| Paternal obesity risk-allele score | Continuous | Linear regression | 31.5 |

BMI, body mass index

*Fully conditional specification method.

**Predicted weight and height were calculated by using previously modelled trajectories from the Jenss–Bayley model (1,2).

1. Carles, S. *et al.* A Novel Method to Describe Early Offspring Body Mass Index (BMI) Trajectories and to Study Its Determinants. *PLOS ONE* **11**, e0157766 (2016).

2. Botton, J. *et al.* Postnatal Weight and Height Growth Modeling and Prediction of Body Mass Index as a Function of Time for the Study of Growth Determinants. *Ann. Nutr. Metab.* **65**, 156–166 (2014).

**Supplementary table S2: Characteristics by quintile of age at AR of the study population.**

|  | **1st quintile N=284** | **2nd quintile N=282** | **3rd quintile N=283** | **4th quintile N=283** | **5th quintile N=283** | **Comparison between quintiles** |
| --- | --- | --- | --- | --- | --- | --- |
|  | **Means (SD) or % (N)** | **Means (SD) or % (N)** | **Means (SD) or % (N)** | **Means (SD) or % (N)** | **Means (SD) or % (N)** | **p*** |
| Sex (boys) | 52.8 (150) | 49.0 (138) | 42.1 (119) | 43.1 (122) | 49.5 (140) | 0.05 |
| Preterm birth | 4.58 (13) | 3.9 (11) | 6.4 (18) | 6.7 (19) | 6.0 (17) | 0.53 |
| Birth weight z-score | -0.26 (1.20) | -0.12 (1.05) | -0.07 (1.01) | -0.05 (1.07) | -0.14 (1.02) | 0.18 |
| SGA | 19.6 (54) | 12.7 (35) | 10.2 (28) | 10.4 (29) | 13.7 (38) | 0.04 |
| AGA | 70.2 (193) | 78.2 (215) | 81.0 (222) | 79.3 (222) | 78.4 (218) | . |
| LGA | 10.2 (28) | 9.1 (25) | 8.8 (24) | 10.4 (29) | 7.9 (22) | . |
| Gestational weight gain (kg) | 13.7 (5.4) | 13.3 (5.0) | 13.2 (4.5) | 13.2 (4.6) | 13.0 (3.8) | 0.52 |
| Maternal BMI (kg/m2) | 24.5 (5.0) | 23.3 (4.7) | 23.0 (4.0) | 22.4 (3.9) | 22.4 (4.1) | 8.4×10^-9^ |
| Paternal BMI (kg/m2) | 26.1 (3.8) | 25.0 (3.7) | 25.2 (3.5) | 24.5 (3.3) | 24.7 (3.4) | 1.2×10^-6^ |
| Never smoking during pregnancy | 76.3 (212) | 73.7 (204) | 78.1 (218) | 82.0 (227) | 83.2 (227) | 0.04 |
| Maternal age at delivery (years) | 30.0 (5.0) | 29.6 (4.8) | 29.5 (4.6) | 30.1 (4.6) | 30.4 (4.6) | 0.09 |
| Maternal educational level (years) | 13.3 (2.4) | 14.0 (2.6) | 14.0 (2.7) | 14.3 (2.6) | 14.3 (2.7) | 4.8×10^-5^ |
| Paternal educational level (years) | 12.8 (2.5) | 13.3 (2.6) | 13.2 (2.7) | 13.5 (2.6) | 13.8 (2.7) | 0.0004 |
| Obesity risk‐allele score | 22.2 (3.4) | 21.7 (3.0) | 21.4 (2.9) | 21.5 (3.2) | 21.3 (3.3) | 0.08 |

* p value of ANOVA for quantitative variable and χ2 for qualitative variable

**Supplementary table S3: Multivariable linear regression analyses of complete cases: factors associated with age at adiposity rebound (days). The EDEN study (N=866)**

| Characteristics | | Model A | |  | Model B | |  | Model C | |  | Model D | |
| --- | --- | --- | --- | --- | --- | --- | --- | --- | --- | --- | --- | --- |
|  |  | **β (SE)** | **P value** |  | **β (SE)** | **P value** |  | **β (SE)** | **P value** |  | **β (SE)** | **P value** |
| Level 1** | Center (ref=Poitiers) | -11.4 (34.6) | 0.74 |  | -22.3 (34.1) | 0.51 |  | -30.5 (34.2) | 0.37 |  | -25,2 (34,2) | 0,46 |
|  | Maternal age at delivery (years) | 2.1 (3.7) | 0.58 |  | 3.2 (3.7) | 0.39 |  | 1.1 (3.8) | 0.77 |  | 1,1 (3,8) | 0,78 |
|  | Maternal educational level (years) | 11.4 (7.8) | 0.15 |  | 8.9 (7.7) | 0.25 |  | 7.0 (7.7) | 0.37 |  | 5,3 (7,7) | 0,49 |
|  | Paternal educational level (years) | 16.4 (7.6) | 0.031 |  | 11.3 (7.5) | 0.13 |  | 11.5 (7.5) | 0.12 |  | 10,8 (7.5) | 0,15 |
| Level 2 | Obesity risk-allele score |  |  |  | -12.6 (5.3) | 0.019 |  | -12.7 (5.3) | 0.017 |  | -12.5 (5.3) | 0.0181 |
|  | Maternal BMI (kg/m^2^) |  |  |  | -15.4 (4.1) | 0.0002 |  | -17.4 (4.2) | 3.3×10^-5^ |  | -16,9 (4,1) | 5.0×10^-5^ |
|  | Paternal BMI (kg/m^2^) |  |  |  | -13.1 (4.9) | 0.008 |  | -12.6 (4.9) | 0.011 |  | -12,7 (4,9) | 0,010 |
| Level 3 | Gestational weight gain (kg) |  |  |  |  |  |  | -10.7 (3.8) | 0.005 |  | -12,6 (3,8) | 0,001 |
|  | Smoking during pregnancy (ref=no) |  |  |  |  |  |  | -31.2 (42.7) | 0.47 |  | -16,0 (42,7) | 0,71 |
| Level 4 | Preterm birth (yes vs no) |  |  |  |  |  |  |  |  |  | -7,4 (85,4) | 0,93 |
|  | Birth weight z-score |  |  |  |  |  |  |  |  |  | 46,1 (16,3) | 0,005 |
|  | Birth weight z-score ^2^ |  |  |  |  |  |  |  |  |  | -20,6 (9,7) | 0,034 |
|  | Sex (boys vs girls) |  |  |  |  |  |  |  |  |  | -45,2 (33,4) | 0,18 |

* Relevance of a variable during the hierarchical regression analyses was determined with the corresponding model in which the variable of interest is first entered, regardless of its performance in the subsequent model(s).

**Supplementary table S4: Linear and logistic regression analyses with a weighted versus unweighted BMI obesity risk-allele score, with multiple imputation or with complete cases**

| Characteristics | N | Linear regression | |  | Logistic regression |
| --- | --- | --- | --- | --- | --- |
|  |  | **β (SE)** | **p value** |  | **OR [95% CI]** |
| Weighted obesity risk-allele score (multiple imputation) | 1415 | -9.3 (3.9) | 0.017 |  | 1.05 [1.00–1.09] |
| Weighted obesity risk-allele score (complete cases) | 866 | -11.1 (4.7) | 0.018 |  | 1.06 [1.01–1.11] |
| Unweighted obesity risk-allele score (multiple imputation) | 1415 | -9.0 (4.5) | 0.047 |  | 1.06 [1.01–1.11] |
| Unweighted obesity risk-allele score (complete cases) | 866 | -12.5 (5.3) | 0.0181 |  | 1.06 [1.01–1.11] |

* Adjusted for center, maternal age at delivery, maternal and paternal educational level, maternal and paternal BMI, gestational weight gain, smoking during pregnancy, preterm birth, birth weight z-score and sex (Model D)

OR, odds ratio; 95% CI, 95% confidence interval

**Supplementary table S5: Multivariable linear and logistic regression analyses with age at adiposity rebound in children born at term (N=1337*)**

| Characteristics | | Linear regression | |  | Logistic regression |
| --- | --- | --- | --- | --- | --- |
|  |  | **β (SE)** | **P value** |  | **OR [95% CI** |
|  | Center (ref=Poitiers) | -16.3 (26.9) | 0.54 |  | 0.97 [0.73–1.29] |
|  | Maternal age at delivery (years) | 4.3 (2.9) | 0.14 |  | 1.01 [0.98–1.04] |
|  | Maternal educational level (years) | 9.8 (6.3) | 0.12 |  | 0.92 [0.86–0.98] |
|  | Paternal educational level (years) | 6.8 (6.1) | 0.27 |  | 1.00 [0.93–1.06] |
|  | Obesity risk-allele score | -9.9 (4.6) | 0.03 |  | 1.06 [1.01–1.11] |
|  | Maternal BMI (kg/m^2^) | -17.3 (3.2) | 9.3×10^-8^ |  | 1.07 [1.04–1.10] |
|  | Paternal BMI (kg/m^2^) | -15.9 (3.9) | 4.2×10^-5^ |  | 1.07 [1.03–1.11] |
|  | Gestational weight gain (kg) | -8.0 (3.1) | 0.01 |  | 1.05 [1.01–1.08] |
|  | Smoking during pregnancy (ref=no) | -69.7 (33.2) | 0.04 |  | 1.04 [0.74–1.47] |
|  | Birth weight z-score (ref=AGA) | - | - |  | - |
|  | SGA | -104.9 (40.6) | 0.01 |  | 2.23 [1.52–3.28] |
|  | LGA | -11.8 (46.3) | 0.80 |  | 1.23 [0.77–1.96] |
|  | Sex (ref=boys) | -35.1 (26.5) | 0.19 |  | 1.32 [1.00–1.75] |

* Missing data for covariates were imputed with the fully conditional specification method.

OR, odds ratio; 95% CI, 95% confidence interval

| 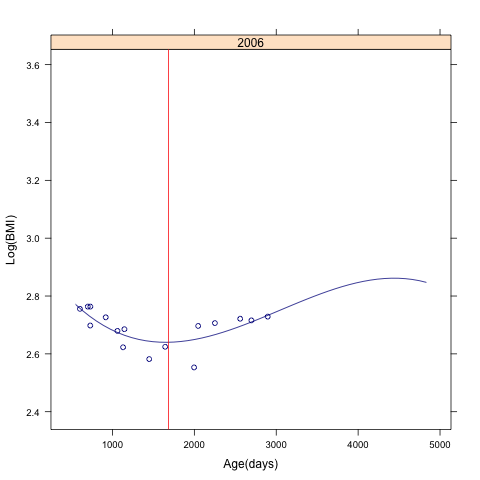 | 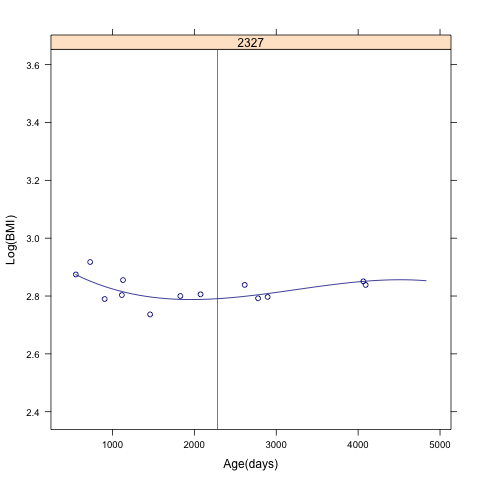 | 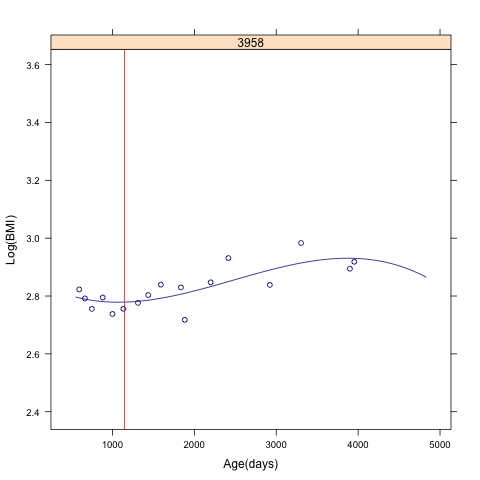 | 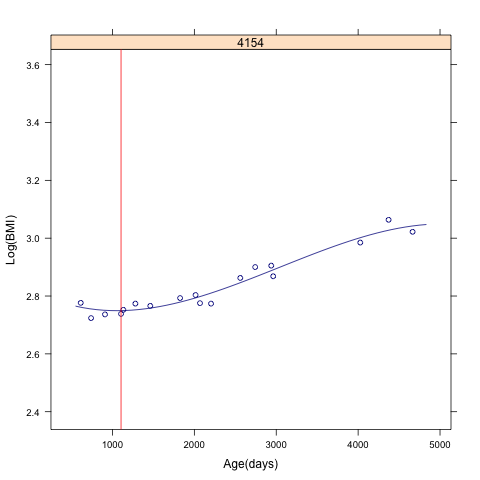 | 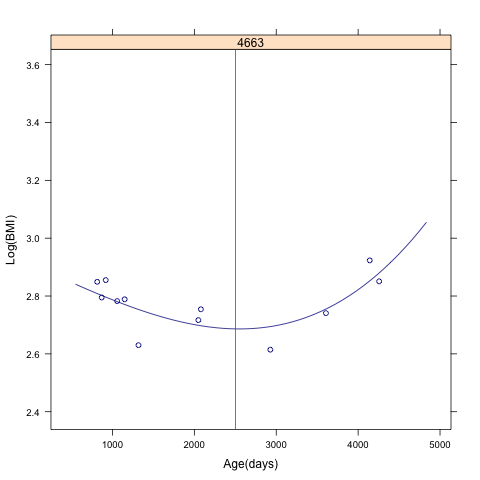 |
| --- | --- | --- | --- | --- |
| 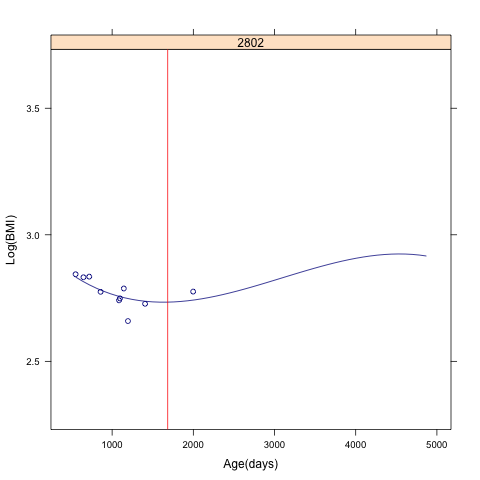 | 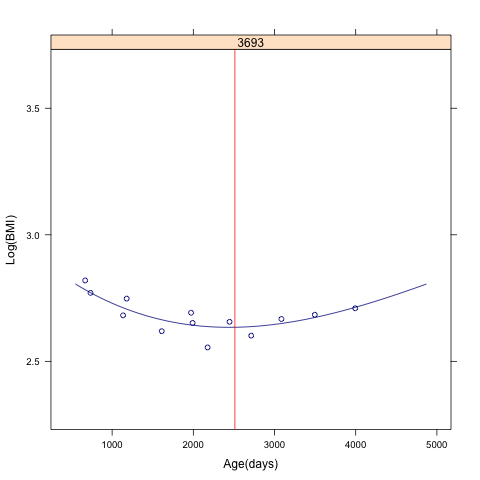 | 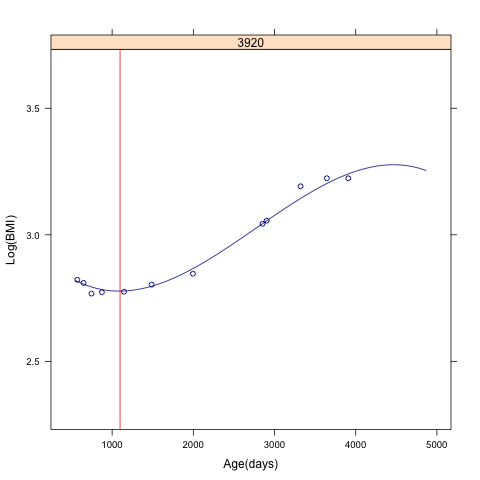 | 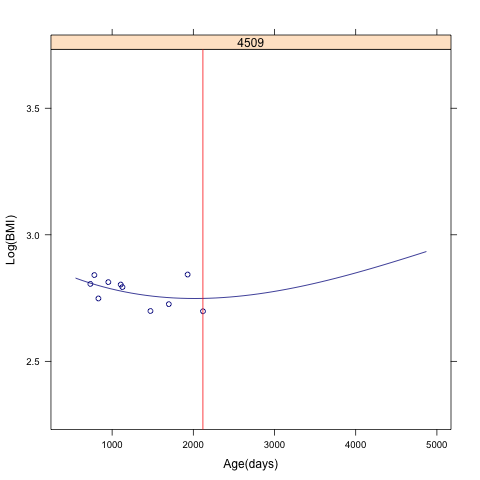 | 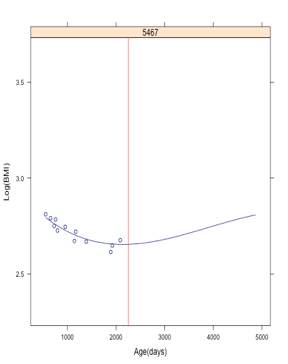 |

**Supplementary figure S1: Modelling results approaches with a random sample of 10 participants.** Points represent the log (BMI) values according to age, blue lines represent individual BMI curves from 18 months to 13 years and red lines the estimated age at adiposity rebound.

| **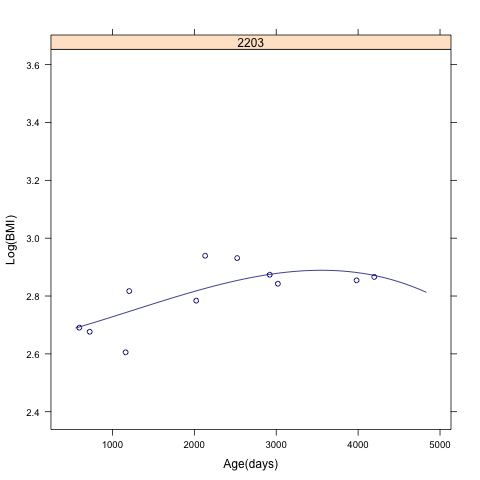** | **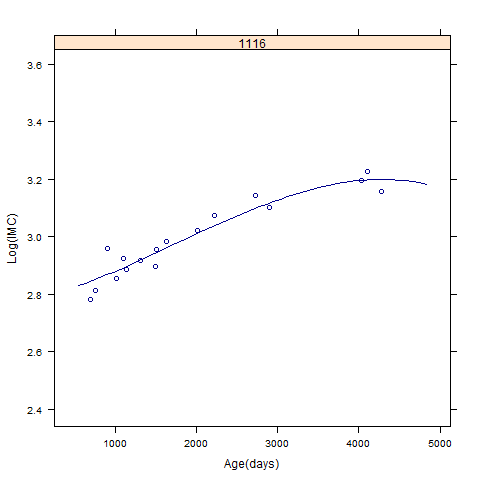** | 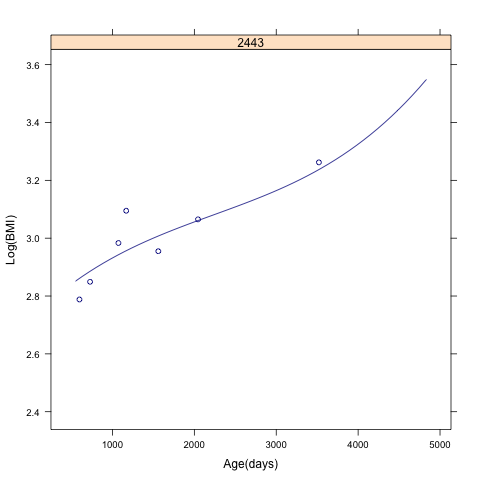 | **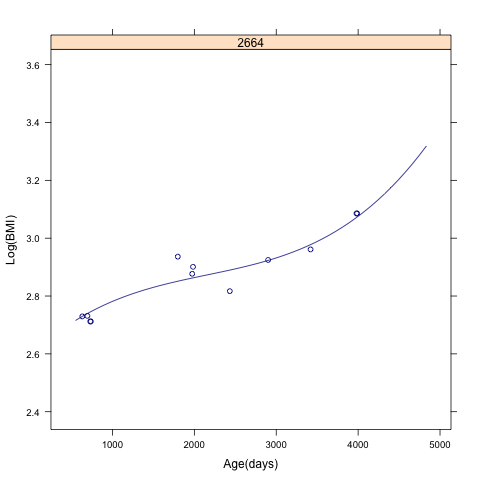** | **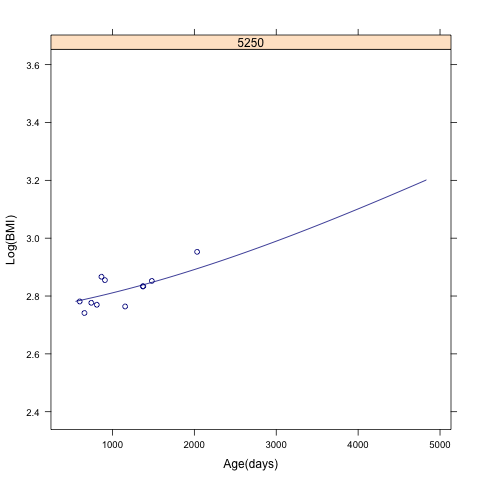** |
| --- | --- | --- | --- | --- |
| **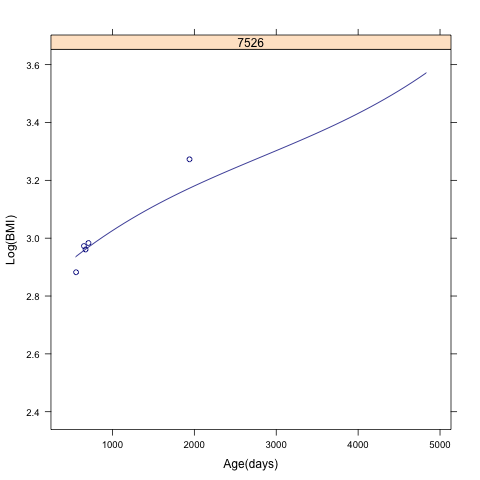** | **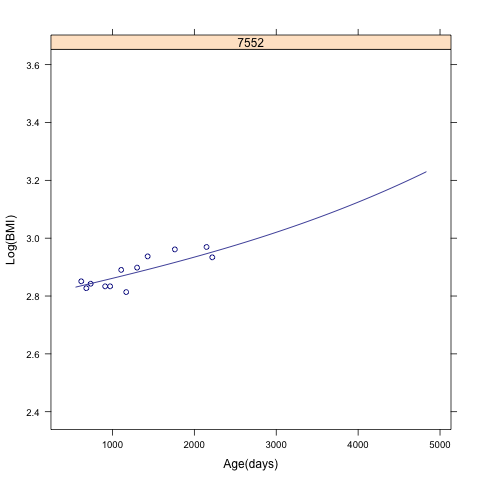** | **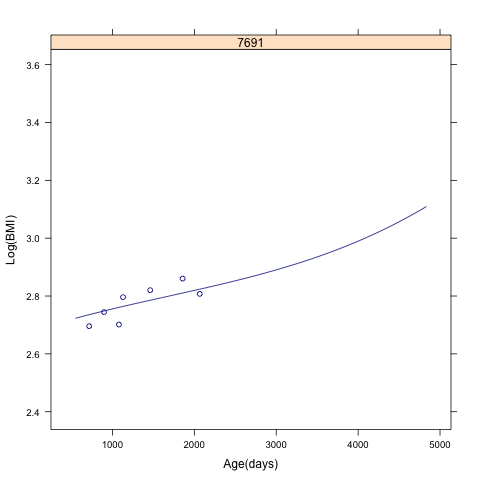** | **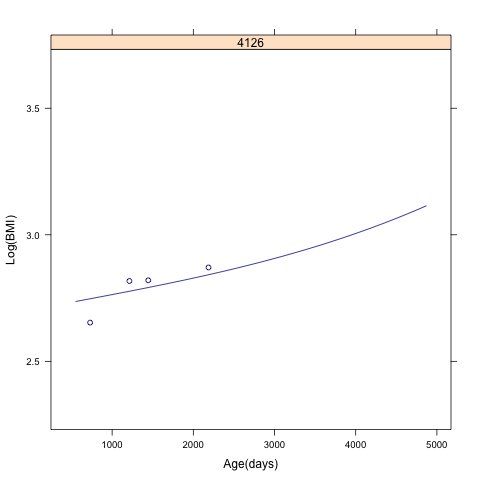** | **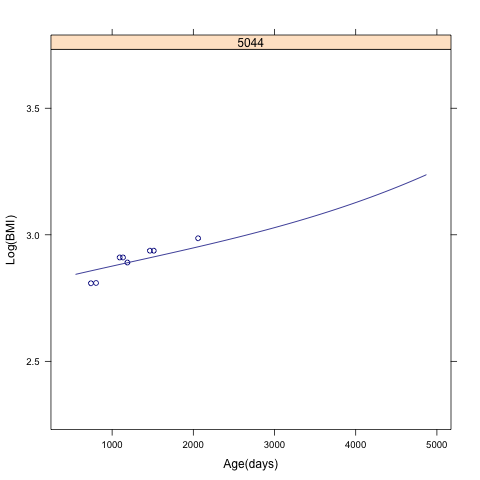** |

**Supplementary figure S2: Modelling results for 10 participants without estimation of adiposity rebound.** Points represent observations of BMI according to age.

Model C

Model B

Model D

Model A

**Demographic and socio-economic factors**

- Recruitment center
- Maternal age at delivery
- Maternal educational level
- Paternal educational level

**Factors related to obesity**

- Child’s obesity risk-allele score,
- Maternal and paternal BMI

**Intrauterine and prenatal environment factors**

- Gestational weight gain `
- Maternal smoking during pregnancy

**Newborn characteristics**

- Sex
- Preterm birth
- Birth weight z-score

**Age at adiposity rebound/ Early adiposity rebound**

**Supplementary figure S3: Conceptual framework of determinants of age at adiposity rebound and early adiposity, prepared for hierarchical logistic and linear regression modeling.** Adapted from Matanda et *al.* with some modification. BMI, body mass index

Matanda DJ, Mittelmark MB, Urke HB, et al. Reliability of demographic and socioeconomic variables in predicting early initiation of breastfeeding: a replication analysis using the Kenya Demographic and Health Survey data. BMJ Open 2014;4:e005194. doi:10.1136/bmjopen-2014- 005194


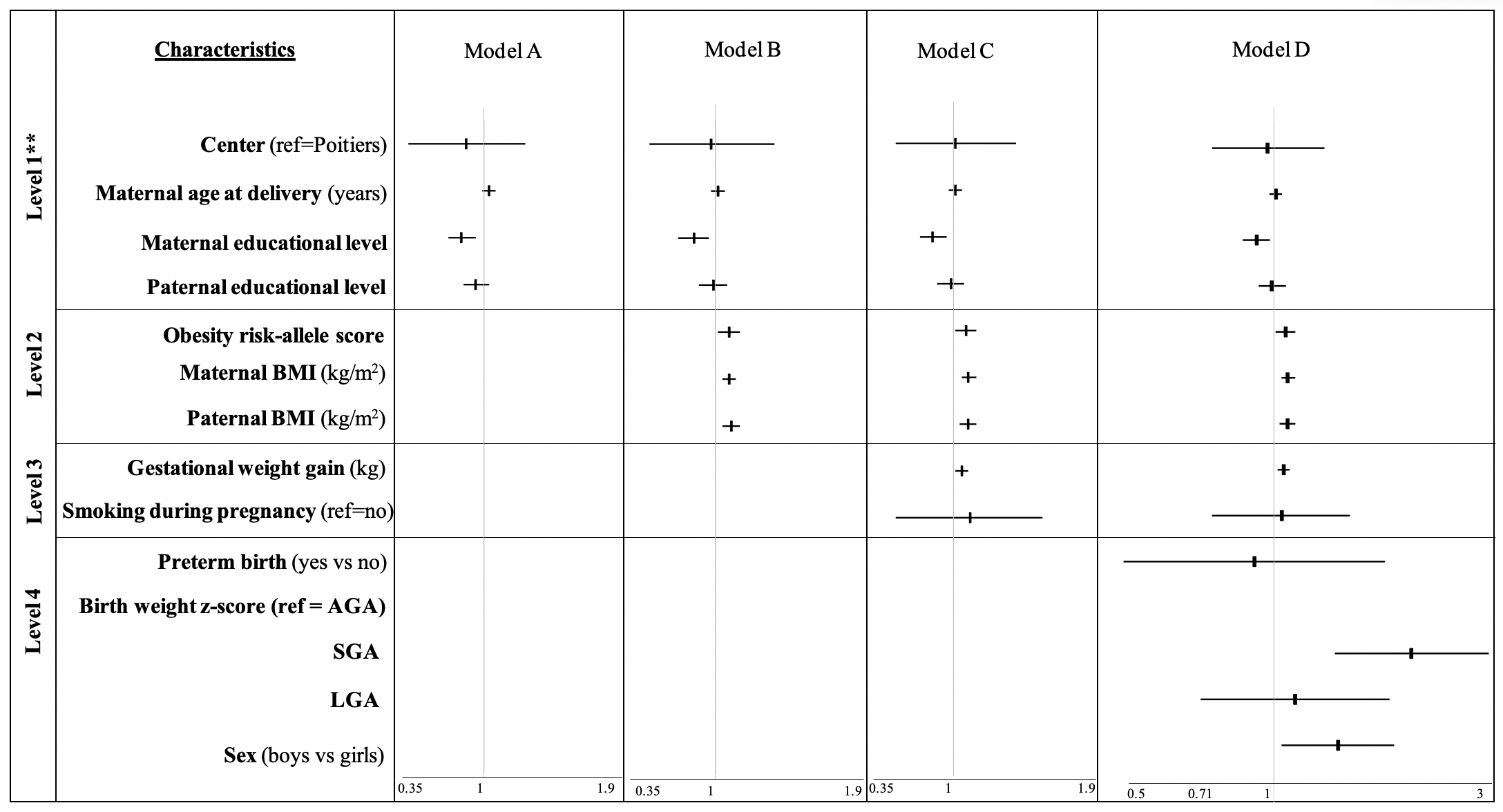


**Supplementary figure S4: Multivariable logistic regression analyses of factors associated with risk of early adiposity rebound defined as being in the lowest quintile of age at adiposity rebound. (N=1415*)**

* Missing data for covariates were imputed with the fully conditional specification method.

**** Relevance of a variable during the hierarchical regression analyses was determined with the corresponding model in which the variable of interest is first entered, regardless of its performance in the subsequent model(s).

| rs10146997 - NRXN3  rs10838738 - MTCH2  rs10913469 - SEC16B  rs11847697 - PRKD1  rs12016871 - MTIF3  rs13107325 - SLC39A8  rs1514175 - TNNI3K  rs1555543 - PTBP2  rs17782313 - MC4R  rs206936 - NUDT3  rs2112347 - FLJ35779  rs2241423 - MAP2K5  rs2287019 - QPCTL  rs2568958 - NEGR1  rs2890652 - LRP1B  rs3810291 - TMEM160  rs4836133 - ZNF608  rs4929949 - RPL27A  rs6548238 - TMEM18  rs9941349 - FTO  rs713586 - RBJ/POMC  rs7138803 - BCDIN3D/F  rs7640855 - CADM2  rs7647305 - TRA2B/ETV5  rs887912 - FANCL  rs925946 - BDNF  rs987237 - TFAP2B | β (in days) |
| --- | --- |

**Supplementary figure S5: Multiple logistic regression analyses for the risk of early age at AR (OR* [95% CI]) on all individual polymorphisms (complete cases analysis, N=799)**

* Adjusted for center, maternal age at delivery, maternal and paternal educational level, maternal and paternal BMI, gestational weight gain, smoking during pregnancy, preterm birth, birth weight z-score and sex (Model D).
